# Chitosan-Hyaluronan Nanoparticles for Vinblastine Sulfate Delivery: Characterization and Internalization Studies on K-562 Cells

**DOI:** 10.3390/pharmaceutics14050942

**Published:** 2022-04-26

**Authors:** Carmela Cannavà, Federica De Gaetano, Rosanna Stancanelli, Valentina Venuti, Giuseppe Paladini, Francesco Caridi, Corneliu Ghica, Vincenza Crupi, Domenico Majolino, Guido Ferlazzo, Silvana Tommasini, Cinzia Anna Ventura

**Affiliations:** 1 Laboratory of Immunology and Biotherapy, Department of Human Pathology, University of Messina, Via Consolare Valeria, 1, I-98125 Messina, Italy; ccannava@alice.it (C.C.); guido.ferlazzo@unime.it (G.F.); 2 Department of Chemical, Biological, Pharmaceutical and Environmental Sciences, University of Messina, Viale Ferdinando Stagno D’Alcontres 31, I-98166 Messina, Italy; fedegaetano@unime.it (F.D.G.); rstancanelli@unime.it (R.S.); stommasini@unime.it (S.T.); 3 Department of Mathematical and Computer Sciences, Physical Sciences and Earth Sciences, University of Messina, Viale Ferdinando Stagno D’Alcontres 31, I-98166 Messina, Italy; vvenuti@unime.it (V.V.); fcaridi@unime.it (F.C.); vcrupi@unime.it (V.C.); dmajolino@unime.it (D.M.); 4 National Institute of Materials Physics, Atomistilor 405A, 077125 Magurele, Romania; cghica@infim.ro

**Keywords:** chitosan/hyaluronan nanoparticles, vinblastine sulfate, transmission electron microscopy, micro-Raman spectroscopy, in vitro studies, K-562 cells

## Abstract

In the present study, we developed chitosan/hyaluronan nanoparticles (CS/HY NPs) for tumor targeting with vinblastine sulfate (VBL), that can be directed to the CD44 transmembrane receptor, over-expressed in cancer cells. NPs were prepared by coating with HY-preformed chitosan/tripolyphosphate (CS/TPP) NPs, or by polyelectrolyte complexation of CS with HY. NPs with a mean hydrodynamic radius (R_H_) of 110 nm, 12% polydispersity index and negative zeta potential values were obtained by a direct complexation process. Transmission Electron Microscopy (TEM) images showed spherical NPs with a non-homogeneous matrix, probably due to a random localization of CS and HY interacting chains. The intermolecular interactions occurring between CS and HY upon NPs formation were experimentally evidenced by micro-Raman (µ-Raman) spectroscopy, through the analysis of the spectral changes of characteristic vibrational bands of HY during NP formation, in order to reveal the involvement of specific chemical groups in the process. Optimized NP formulation efficiently encapsulated VBL, producing a drug sustained release for 20 h. In vitro studies demonstrated a fast internalization of labeled CS/HY NPs (within 6 h) on K-562 human myeloid leukemia cells. Pre-saturation of CD44 by free HY produced a slowing-down of NP uptake over 24 h, demonstrating the need of CD44 for the internalization of HY-based NPs.

## 1. Introduction

The treatment of malignancies is currently based on the use of cytotoxic chemotherapy drugs, whose action is designed to directly damage the DNA or to inhibit cell replication in a non-specific manner, causing the death of both tumor cells and normal cells in the replication phase. This inability of anticancer drugs to adequately distinguish malignant cells from healthy ones represents a major limitation in the treatment of tumors. For this reason, new therapeutic approaches aimed at improving the selective delivery of drugs in tumors are of increasing interest. In this context, different delivery systems to the tumor cells such as micelles [1] and liposomes [2] were studied, and polymeric nanoparticles (NPs) received particular attention for their ability to protect the encapsulated drug from chemical and physical degradation, to produce the sustained release of the loaded molecules and to deliver the drugs to specific tissues or organs [3,4,5]. Polymeric NPs prepared by ionotropic gelation of polyelectrolytes received significant attention as systems for controlled drug release and targeting. Their simple preparation process, which avoids the use of both organic solvents and surfactants, together with their ability to encapsulate both macromolecules and small hydrophilic molecules, make them very interesting for drug delivery [6,7]. Chitosan (CS), consisting of copolymers of N-acetyl-D-glucosamine and D-glucosamine units linked by β-(1,4)-glycosidic linkages, is a hydrophilic linear polysaccharide characterized by low toxicity and high biodegradability and biocompatibility [8,9]. CS has been shown to be degraded mainly by lysozyme [10], which commonly exists in various human bodily fluids and tissues. As a result of the existence of amine groups, CS is positively charged at neutral or acidic pH and it is able to form intermolecular complexes with a wide variety of polyanions, such as tripolyphosphate (TPP), sodium sulphate and, as recently reported, also with negatively charged cyclodextrins [11,12,13]. However, these NPs typically exhibit a cationic surface, which determines a quick and unselective cellular uptake, mainly in phagocytic cells [14]. Interaction of CS with other polyanionic macromolecules, including hyaluronan (HY) [15], produces NPs with modified surfaces and consequently different body distributions [16]. In particular, polyelectrolytic complexation of CS with HY is very important to obtain NPs that target specific cells [17]. HY is a linear polysaccharide composed of repeating disaccharide units of D-glucuronic acid and N-acetyl glucosamine linked by β-(1,4) and β-(1,3) glycosidic bonds. It is a natural polymer that is internalized by cells by means of active transport by transmembrane glycoprotein *Cluster of Differentiation 44* (CD44) [18]. These receptors are present in a great variety of cells, such as macrophages and lymphocytes, but are over-expressed in many types of cancer cells [19], hence representing a suitable marker for tumor targeting. Therefore, delivery systems that expose HY on the surface can increase intracellular drug accumulation, specifically in CD44 over-expressing cancer cells [20,21,22]; this can avoid serious side effects related to cancer therapy. Recently, Lallana et al. [23] realized CS/HY NPs that efficiently deliver a large luciferase-encoding mRNA and a much smaller anti-Luc siRNA into cells overexpressing CD44 receptors. These systems showed optimal performance under slightly acidic conditions, typical of tumoral extracellular environments, demonstrating great potential of this cargo to treat cancerous disease. Similarly, Choi et al. [24] demonstrated high intracellular uptake of self-assembled fluorescent HY-NPs within SCC7 cancer cells over-expressing CD44. Instead, no significant fluorescence signals were observed in the cytosol when the labeled NPs were incubated with normal fibroblast cells (CV-1), or with excess HY-free treated SCC7 cells.

Vinblastine sulphate (VBL) is an alkaloid drug that shows cytotoxic activity. It arrests cell growth at the metaphase by binding to the microtubular proteins of the mitotic spindle, preventing polymerisation [25]. The drug was approved since 1961 by the Food and Drug Administration as a pharmaceutical strategy against different tumour types such as leukemia, Hodgkin’s lymphoma, non-small cell lung cancer, breast cancer and others [26]

Even if its action is more pronounced on the rapidly dividing cells than on the normal cells, VBL presents serious debilitating toxic manifestations typical of anticancer agents, and among them, neutropenia is the principal dose-limiting side effect [27,28]. Mucositis, nausea, vomiting and headaches represent the most common adverse effects. For this reason, the development of alternative strategies able to direct the drug to cancer cells, reducing adverse effects of VBL and increasing its potential anticancer activity is needed. Some nanocarriers for VBL delivery are already described in the literature [29,30,31], where the conjugation of the carrier with folate was used as a strategy to obtain formulations with active targeting properties. To our knowledge, no papers exist that describe the potentiality of CS/HY NPs for VBL delivery in cancer cells.

On these bases, we developed CS/HY NPs, able to interact with CD44-transmebrane receptors, for selective delivery of VBL to cancer cells. The NPs were prepared using ionotropic gelation of CS with TPP and subsequent coating with HY (CS/TPP-HY NPs), or by a direct polyelectrolyte complexation of the polycation with HY (CS/HY NPs). They were comparatively characterized for size, surface electric properties and morphology. Furthermore, a detailed study of the NPs internal architecture was performed by Raman measurements, to detail how HY and CS interact with each other via hydrogen bonding (HB). The optimized NP formulation was loaded with the anticancer drug VBL, and the encapsulation parameters and release rate were evaluated. Furthermore, the in vitro cytotoxicity of VBL-loaded NPs was assayed, and compared to free VBL on K-562 cell line (human chronic myelogenous leukemia cells). In order to demonstrate the efficacy of CS/HY NPs in tumoral targeting, NPs were labeled by encapsulating a labeled bacterial lysate of *Lactobacillus reuteri* and in vitro internalization studies on K-562 cell line were performed by flow cytometry and ImageStream flow cytometry.

## 2. Materials and Methods

### 2.1. Materials

Hyaluronan (Novozymes Hyasis^®^; HY, MW = 0.6–1.1 MDa) was kindly supplied by Novozymes Biopharma (Krogshoejvej 36, 2880 Bagsvaerd, Denmark). Chitosan (CS) low molecular weight (75–85% deacetylated; MW, 50.000–190.000 Da), vinblastine sulfate (VBL), sodium tripolyphosphate (TPP), PKH26 Red Fluorescent Cell Linker and Diluent C were purchased from Sigma-Aldrich (Sigma-Aldrich, Darmstadt, Germany). Human chronic myelogenous leukemia cell line (K-562—ATCC^®^-CCL-243), CD44-negative human breast cancer lines (ZR-75-1—ATCC CRL-1500), Trypsin- 0.53 mM EDTA solution and *Lactobacillus reuteri* (Kandler et al.—ATCC PTA-6475) (*L. reuteri*) were furnished by American Type Culture Collection (Rockville, MD, USA). The RPMI 1640 culture medium, supplemented with fetal bovine serum (FBS, 10% *v/v*) and bovine serum albumin (BSA), were furnished by the EuroClone company (Milan, Italy). Alexa fluor 647 anti-human CD107a (LAMP-1) antibody, FITC anti-mouse/human CD44 and FITC Human IgG2 Isotype Control Recombinant Antibody (FITC-γ2b) were purchased from BioLegend (San Diego, CA, USA). The water used throughout the study was “Lichrosolv” HPLC ultrapure water (MERCK KGaA, Darmstadt, Germany). All other products and reagents were of analytical grade.

### 2.2. Hydrolysis of Hyaluronic Acid

Before use, HY was subjected to a hydrolysis reaction to fragment the chains and obtain low molecular weight HY. The hydrolysis was carried out following a slightly modified method reported in the literature [32]. Briefly, 500 mg of HY was solubilized in 50 mL of distilled water, and the pH was brought to 2 by means of HCl solution addition (1 M). The solution was maintained under magnetic stirring (300 rpm) at 60.0 ± 0.5 °C for 24 h, then brought to pH 7 by adding 1 M NaOH drops and transferred into a dialysis tube (Spectra/Por3 Dialysis Membrane, MWCO = 3500). It was dialyzed against water for 4 days, while changing the dialysis medium every 24 h. The dialysate was then centrifuged (ThermoFisher Scientific, Waltham, MA, USA, Heraeus megafuge 16) at 5000 rpm for 15 min, and the supernatant containing the hydrolyzed polymers was freeze-dried (VirtTis Benchtop K Instrument, SP Scientific, Gardiner, NY, USA).

### 2.3. Purification of Chitosan

CS was purified following a method reported in literature [33]. Briefly, 5 g of CS were dissolved in acetic acid solution (400 mL, 2% *v/v*) under magnetic stirring. The obtained solution was kept boiling for 15 min, to denature and precipitate the protein contaminants, then centrifuged for 10 min at 4500 rpm (ThermoFisher Scientific, Heraeus megafuge 16). The pellet was discarded, and the supernatant withdrawn and filtered through 0.45-micrometer Millipore^®^ GSWP filters (Bedford, MA, USA). CS was precipitated from the filtrate by adding NaOH solution (1 N) until pH 9 was reached. After centrifugation of the suspension at 5000 rpm for 20 min, the pellet constituted by CS was redispersed in aqueous solution at pH = 9 and then re-separated by centrifugation. The supernatant was discarded, while the pellet was collected and freeze-dried for 72 h (VirtTis Benchtop K Instrument, SP Scientific, Gardiner, NY, USA).

### 2.4. Preparation of Labelled Bacterial Lysate

*L. reuteri* was labeled with PKH26 according to the following procedure: two solutions were prepared, one consisting of 100 μg of bacteria in 1 mL of diluent C and the second consisting of 2 μL PKH26 in 1 mL of diluent C. The two solutions were combined and left for 5–7 min at room temperature, then a wash with supplemented RPMI was performed to inactivate the reaction. The suspension was centrifuged at 4500 rpm for 20 min at 4 °C (3-16PK Sigma Centrifuge, Sigma Laborzentrifugen GmbH, Osterode am Harz, Germany). The supernatant was removed, and the pellet re-suspended in 500 μL of supplemented RPMI. The lysis of the labeled bacteria was then performed by subjecting them to 4 sonication cycles (Elmasonic S 30 (H), Elma Schmidbauer GmbH, Singen, Germany) of 30 min each, in ice bath, using a frequency of 37 kHz and an intensity of 14 W/cm^2^.

### 2.5. Preparation of CS NPs Gelled with TPP and Coated with HY

CS NPs gelled with TPP (CS/TPP NPs) were obtained using different amounts of the two components while maintaining a constant total volume (5 mL). The compositions of the individual formulations are reported in Table 1.

CS was solubilized, under constant magnetic stirring in 4 mL of 1% (*v/v*) acetic acid solution; TPP was solubilized in 1 mL of water. Both solutions were then brought to pH 5 by addition of 2 M NaOH drops, and filtered through 0.22-micrometer Millipore^®^ GSWP filters (Bedford, MA, USA). CS/TPP NPs were obtained by slowly dripping the TPP solution into the polycation solution. The opalescent solution obtained was maintained for 30 min under constant magnetic stirring, then placed in an ultrasonic bath for 15 min, then left to rest overnight. The CS/TPP NPs colloidal suspension was then centrifuged at 5000 rpm for 5 min (ThermoFisher Scientific, Heraeus megafuge 16) to eliminate the CS aggregates that eventually were formed. The pellet was discarded while the supernatant was centrifuged at 13,000 rpm for 45 min. The supernatant was eliminated and the pellet, consisting of CS/TPP NPs, was re-dispersed in 2 mL of water and dripped into an aqueous solution of HY (0.5 mg/20 mL) previously brought to pH 5 by adding drops of 2 M NaOH and filtered as performed for the other polymers. The colloidal suspension was maintained under constant magnetic stirring for 1 h, then centrifuged at 13,000 rpm for 45 min. The pellet containing CS/TPP NPs coated with HY (CS/TPP-HY) was recovered and re-dispersed in 1 mL of water for the chemical-physical characterization.

### 2.6. Preparation of CS NPs by Polyelectrolyte Complexation with HY

For CS/HY NP preparation, 2.82 mg of CS were solubilized in 4 mL of 1% (*v/v*) acetic acid solution while HY (0.56 mg) was solubilized in 2.0 mL of water. The polymeric solutions were filtered through 0.22-micrometer Millipore^®^ GSWP filters (Bedford, MA, USA) and brought to pH 5 by adding 1 M HCl (to CS solution) or 1 M NaOH (to HY solution). After that, HY solution was dripped slowly, and under constant magnetic stirring, into the polycation solution. The obtained colloidal suspension was left to stir for about 30 min and centrifuged first at 5000 rpm at 25 °C for 15 min (ThermoFisher Scientific, Heraeus megafuge 16) to remove eventual larger particles present as pellet. The supernatant was then centrifuged at 25,000 rpm at 4 °C for 30 min (3-16PK Sigma Centrifuge). The supernatant was discarded while the pellet was re-suspended in 1 mL of water for chemical-physical characterization.

### 2.7. Preparation of CS/HY NPs Loading VBL and CS/HY NPs Loading Labeled L. reuteri

CS/HY NPs loading VBL were prepared using the same procedure used for the preparation of empty CS/HY NPs, adding different amounts of the drug (5, 15 and 20% *w/w* in polymers) to CS solution. At the end of the procedure, the pellet containing VBL loaded-NPs was collected, resuspended in 1 mL of water containing 5% (*w/v*) trehalose as cryoprotectant, and then freeze-dried for successive chemical-physical characterization. The supernatant was filtered (LLG-Syringe filters Spheros, Meckenheim, Germany, 0.22 µm Ø 25 mm) and the amount of VBL present was estimated by UV–vis spectroscopy in the 200–400 nm spectral range, at 25.0 ± 0.1 °C, by using a FullTech Instruments (mod. PG T80, Rome) for the indirect determination of the encapsulation efficiency (E.E.) according to the following:

E.E. (%) = [(Theoretical VBL in mg − VBL in supernatant in mg)/Theoretical VBL in mg] × 100,(1)

For the preparation of CS/HY NPs loading labelled *L. reuteri* (labeled CS/HY NPs), the bacterial lysate was added, drop by drop, to HY solution. Then, labeled NPs were prepared using the same procedure described for the preparation of empty CS/HY NPs. At the end of the process, the pellet containing labeled CS/HY NPs was re-dispersed in 500 μL of supplemented RPMI 1640, and used for the internalization assays.

### 2.8. Determination of Sizes and Surface Electrical Properties of NPs

The mean hydrodynamic radius of NPs was determined via photon correlation spectroscopy (PCS) by a Zetasizer Nano ZS (Malvern Instrument, Malvern, UK), utilizing a noninvasive back-scattering (NIBS) technique. The measurements were performed at a 173° angle with respect to the incident beam at 25 ± 1 °C for each dispersion. The deconvolution of the measured correlation curve to an intensity size distribution was achieved by using a non-negative least-squares algorithm.

Zeta potential (ζ) of NPs was determined using a Zetasizer Nano ZS instrument (Malvern Instruments Ltd., Malvern, UK) equipped with a He-Ne laser, 5 mW, operating at 633 nm. A suitable quantity of each sample (100 μL) was appropriately diluted with 20 mL of water, and injected into the electrophoretic cell of the instrument. The ζ was determined on the basis of electrophoretic mobility using the Smoluchowsky equation and a nominal value of the Smoluchowsky constant equal to 1.5. Each reported value is an average of 10 experiments.

### 2.9. Morphological Characterization of NPs

The morphology of the samples was investigated by TEM measurements performed at a high resolution transmission electron microscopy (HRTEM) facility at the National Institute of Material Physics in Bucharest (Romania), using a JEOL ARM200F Analytical Transmission Electron Microscope (JEOL Ltd., Tokyo, Japan). A drop of the NP suspension was mounted on a copper grid with a holey carbon film, and the solvent was allowed to evaporate at room temperature for 12 h. After that, samples were ready for TEM measurements. In order to elucidate the surface distribution of the HY in the NPs, CS/HY NP colloidal suspensions were incubated with 10-nanometer Au nanoparticles for 1 h. After this treatment, the NPs were deposited on TEM grids and dried for 12 h before the TEM measurements.

### 2.10. Molecular Characterization of Chemical and Physical Interactions in NPs

µ-Raman measurements were carried out on HY, CS and CS/HY NP systems at solid state by means of a “BTR 111 Mini-RamTM” (B&W Tek, Inc., Newark, NJ, USA) portable spectrometer, using an excitation wavelength of 785 nm (diode laser), 280 mW maximum laser power at the excitation port, and a charge-coupled device (CCD) detector (thermoelectric cooled, TE). The laser output power can be continuously adjusted in order to achieve the best signal-to-noise ratio in the minimum integration time. Spectra were acquired in the 700–1800 cm^−1^ range using a resolution of 10 cm^−1^, and an acquisition time of 10 s × 32 scans. A calibration procedure was applied by means of the peak of a silicon chip at 520.6 cm^−1^, in order to ensure the best instrument performance. The system was equipped with a BAC151B Raman microscope. An 80× objective was used, with a working distance of 1.25 mm and laser beam spot size of 26 µm. The maximum power delivered to the samples was ~120 mW.

### 2.11. In Vitro VBL Release from NPs

The in vitro release of VBL from NPs was evaluated using dynamic Franz diffusion cells (LGA, Berkeley, CA, USA), with a diffusional surface area of 0.75 cm^2^ and a volume of 4.75 mL of the receptor compartment. A synthetic cellulose membrane (molecular cut-off 8000 Da) was placed between the donor and receptor compartments. Free VBL (0.5 mg) and the same amount of CS/HY NPs were poured in 500 µL of phosphate buffer solution (PBS, pH 7.4 and pH 5.0), and an aliquot of 200 µL of each sample was placed in the donor chamber. The receptor compartment was filled with PBS (pH 7.4) and was maintained at 37.0 ± 0.1 °C under continuous stirring. At fixed times (1, 3, 5, 8, 10, 20 and 24 h), 500 µL of each sample was collected from the receptor compartment and analyzed by UV-vis spectroscopy, using the previously reported apparatus. The collected volume was replaced by fresh PBS (pH 7.4). The experiments were conducted in triplicate and the results were expressed as means ± S.D.

### 2.12. In Vitro Biological Assays

#### 2.12.1. Cell Cultures

The K-562 cell line was grown in flasks using RPMI 1640 culture medium supplemented with 10% *v/v* FBS, 2 mM glutamine, 100 U/mL penicillin and 100 μg/mL of streptomycin (GIBCO, Invitrogen Corporation, Paisley, UK). Cells were cultivated in a standard incubator for cell cultures (Thermo Fisher Scientific, Rome, Italy), with humidified air at 37.0 ± 0.5 °C with 5% of CO_2_, 70% humidity, at a concentration between 0.3 × 10^6^ and 1.5 × 10^6^ cells/mL. The cells were kept alive by means of steps taken 2 or 3 times a week, consisting of centrifugation of the cell suspension at 1600 rpm at 4 °C for 6 min (3-16PK Sigma Centrifuge), and resuspension of the pellet obtained in adequate volume of complete soil. The estimate of cell viability was carried out by staining with Trypan Blue (GIBCO, Invitrogen Corporation, Paisley, UK) in a Bürker counting chamber. For each experiment, 5 × 10^5^ cells were used. CD44 surface expression on K-562 cells was verified using FITC anti-mouse/human CD44. Two tubes were prepared containing 5 × 10^5^ cells each, washed from the culture medium with PBS (pH 7.4) and centrifuged at 1600 rpm for 6 min at 4 °C (3-16PK Sigma Centrifuge). Once the supernatant was removed, in one of the tubes 5 μL of FITC anti-human CD44 antibody were added, whereas in the other tube the isotypic control was performed with FITC-γ2b to exclude non-specific fluorescence. The samples were incubated for 30 min in the dark at 4 °C; the excess antibody was then removed by washing three times with PBS (pH 7.4) and centrifuging at 1600 rpm for 6 min at 4 °C (3-16PK Sigma Centrifuge). The sample, re-suspended in a few μL of PBS (pH 7.4), was analyzed by acquiring 10^4^ events by flow cytometer (BD FACSCanto II Flow Cytometer, Marshall Scientific, Hampton).

The CD44-negative ZR-75-1 cell line [34] was grown in flask using RPMI 1640 culture medium supplemented with 10% *v/v* FBS, 2 mM glutamine, 100 U/mL penicillin and 100 μg/mL of streptomycin (GIBCO, Invitrogen Corporation, Paisley, UK), in a standard incubator for cell cultures (Thermo Fisher Scientific, Rome, Italy) with humidified air at 37.0 ± 0.5 °C with 5% of CO_2_, 70% humidity. When the cells were approximately 50% confluent, the medium was removed and the cells were washed twice in PBS (pH 7.4). The cells were trypsinized by adding 2 mL of 0.25% (*w/v*) Trypsine-0.53 mM EDTA solution to remove all traces of serum that contained trypsin inhibitor, and left for 4 min in a standard incubator for cell cultures (Thermo Fisher Scientific, Rome, Italy) (humidified air at 37.0 ± 0.5 °C, 5% of CO_2_, 70% of humidity). After that, 10 mL of PBS (pH 7.4) were added to the trypsinized cells; the cells were counted, and a cellular suspension in supplemented RPMI at a concentration of 5 × 10^5^ cells/mL was prepared. The cells were seeded in Tranwell^®^-Clear Insert, Polyester Membranes, and then 1.5 mL of supplemented RPMI was added.

#### 2.12.2. Cell Internationalization Studies

To verify the ability of CS/HY NPs to be internalized by CD44-overexpressed K-562 and CD44-negative ZR-75-1 cells, analyses were performed using Flow Cytometry (BD FACSCanto II Flow Cytometer, Marshall Scientific, Hampton, VA, USA) and ImageStream Flow Cytometry (Amnis^®^ ImageStream^®X^, Inspire TM software).

Several cell samples were prepared:

1. K-562 cells supplemented with unlabeled CS/HY NPs (negative control) or labeled CS/HY NPs;
2. K-562 cells pretreated with an excess of HY (10 times higher than that used for the preparation of the NPs), left to incubate for 1 h, washed with PBS (pH, 7.4), and then supplemented of labeled CS/HY NPs;
3. K-562 cells supplemented with unlabeled CS/TPP NPs (negative control) or labeled CS/TPP NPs;
4. K-562 cells in RPMI 1640 culture medium (control);
5. ZR-75-1 cells supplemented with unlabeled CS/HY NPs (negative control) or labeled CS/HY NPs;
6. ZR-75-1 cells in RPMI 1640 culture medium (control).

All samples were kept in an incubator under standard growth conditions (overnight in thermostat at 37.0 ± 0.5 °C, 5% CO_2_), then centrifuged (1600 rpm, 6 min, 4 °C). The pellets were resuspended in 200 μL of PBS (pH 7.4), and cytofluorimetric analysis was performed at three different time points (1 h, 6 h and 24 h).

Samples containing the unlabeled NPs were prepared to exclude any auto cell fluorescence signal not linked to the internalization of the NPs. The sample pretreated with excess HY was prepared in order to saturate the CD44 membrane receptor and to clarify whether the mechanism of internalization of the NPs was associated to their interaction with this receptor.

#### 2.12.3. Intracytoplasmic Staining of K562 Cells

The K-562 cells cultured in supplemented RPMI 1640 were washed with PBS, and a fixation was made in paraformaldehyde solution (PFA, 2%, *w/v*); then, another washing was performed in PBS (pH 7.4) and the permeabilization of the cells in BSA 0.5% (*v/v*) and saponin 0.1% (*w/v*) was carried out. The cells were labeled with Alexa fluor 647 anti-human CD107a (LAMP-1) antibody (10 μL of a solution 1:10 in BSA 0.5%, *v/v*, and saponin 0.1%, *w/v*) for 15 min at 37 °C. The experiment was performed on K-562 cells treated for 6 h with the NPs loading labeled and not labeled *L. reuteri* bacterial lysate.

#### 2.12.4. Evaluation of Cytotoxic Activity

The cytotoxic activity of 15% VBL-CS/HY NPs was evaluated in comparison with the free drug by MTT assay. K-562 cells were seeded into 96-well cell culture plates (5 × 10^3^/0.66 cm^2^) and incubated at 37 °C. After 24 h of incubation, the cells were treated with different concentrations (25, 50, 100 μg/mL) of free VBL, or with the same amount encapsulated within CS/HY NPs, for 12 and 24 h. Empty CS/HY NPs were assayed for the same times at a polymer concentration corresponding to that present in the VBL-CS/HY NPs used. Untreated cells were used as the control during experiments. MTT solution (0.5% in PBS, pH 7.4) was added to each well and further incubated for 4 h. The cell culture medium was then removed, and the obtained formazan crystals precipitated on the bottom of well were dissolved using 100 μL of a DMSO. The optical density of each sample was measured using a microplate spectrophotometer (Titertek Multiskan; DAS, Milan, Italy) set to a wavelength of λ = 550 nm. Data are the average of three different experiments (6 replicates for each point) ± standard deviation.

#### 2.12.5. Statistical Analysis

One-way ANOVA testing was carried out to evaluate statistical significance. A Bonferroni *t*-test analysis was used to validate the ANOVA test. A value of *p* < 0.05 was considered as the minimal level of significance in the various experiments.

## 3. Results and Discussion

### 3.1. Dimensional and Morphological Characterizations of NPs

The size of NPs for parenteral administration plays an important role in determining the biodistribution and internalization of NPs in *target* cells. They must be small enough (<200 nm) to not be recognized as foreign bodies by macrophages, and to circulate freely within the circulatory stream; at the same time, the NPs must not be so small (>30 nm) in order to be eliminated through the urinary tract before reaching the *target* tissue [35].

To identify the optimal CS/TPP NP formulation in terms of sizes and electrical surface characteristics suitable for the successive coating with HY, we prepared systems in which the CS/TPP weight ratio was changed while the volume was maintained unaltered, as reported in Table 1 of the Materials and Methods section. Among all the prepared formulations, only the one with a CS/TPP weight ratio of 4:1 (sample 1) was found to have suitable characteristics in terms of average sizes, polydispersity index (P.I.) and zeta potential (ζ), to continue with the coating with HY (see sample 1 in Table 2); the other preparations underwent flocculation due to an excess of one of the two reagents, or because they produced very low yields (data not shown). The coating of CS/TPP NPs with HY seems to give rise to a system with slightly lower average sizes, but with a high polydispersity index indicative of high heterogeneity of the system (see sample 2 in Table 2).

**Figure 1:**
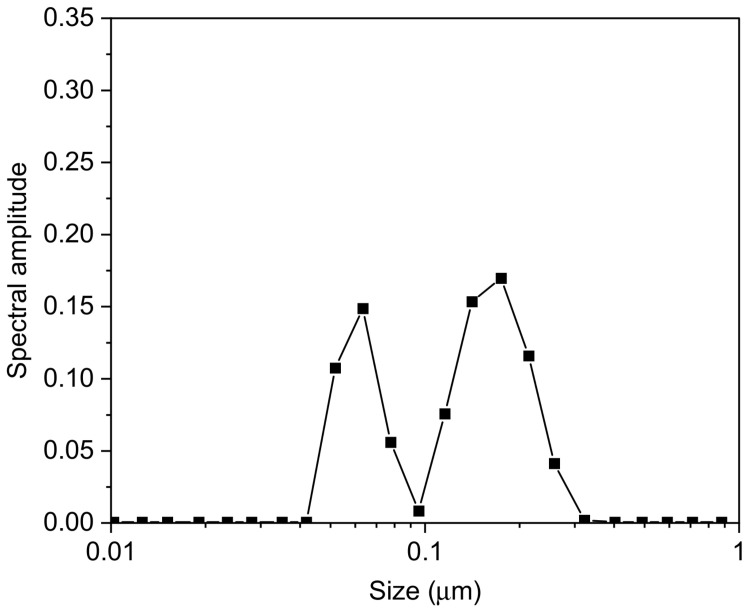
Dimensional analysis of CS/TPP-HY NPs.

As shown in Figure 1, the colloidal dispersion consisted of two populations, the first one having R_H_ around 80 nm, and the other one with R_H_ of about 230 nm. 

As reported by Gennari et al. [36] during the coating of CS/TPP NPs with HY, two processes could take place—a coating of NPs with HY and/or an exchange between TPP present into the matrix and HY. On this basis, the largest population is presumably due to a superficial complexation of CS/TPP NPs with HY chains, with a consequent increase in size. The smallest population could be the result of polyanion exchange; during this process, a new arrangement of the matrix could take place with a consequent reduction in size. It cannot be excluded that the larger population is the result of an aggregation of the smaller nanoparticles. In all cases, the presence of HY on the surface was evidenced by the inversion of the ζ value from positive, due to the presence on the NP surface of CS amino groups, to negative, due to the superficial exposure of negative HY (Table 2).

Polyelectrolyte complexation between CS and HY was obtained by contact of the two polymers at a concentration established by our previous preformulation studies, which produced NPs with very low sizes and high homogeneity (see sample 3 in Table 2).

The ζ value of this system turned out to be less negative with respect to the value determined for NPs obtained by coating with HY. This result is probably a result of the different nature of the NPs produced with the two methods. Reasonably, the ionotropic gelation and successive HY coating produced NPs with a more negative charge density on the surface as a result of the homogeneous presence of adsorbed HY. On the other side, when direct complexation is used for the preparation of the NPs, the architecture of the matrix is made of interacting CS and HY chains. This will produce a surface charge distribution that is expected to be non-uniform, due to the presence of CS-rich (positive charged) and/or HY-rich (negative charged) areas randomly distributed.

As a result of the good properties, particularly in terms of size shown by NPs prepared by polyelectrolyte complexation of CS and HY, we decided to select these NPs to carry out all successive studies.

**Figure 2:**
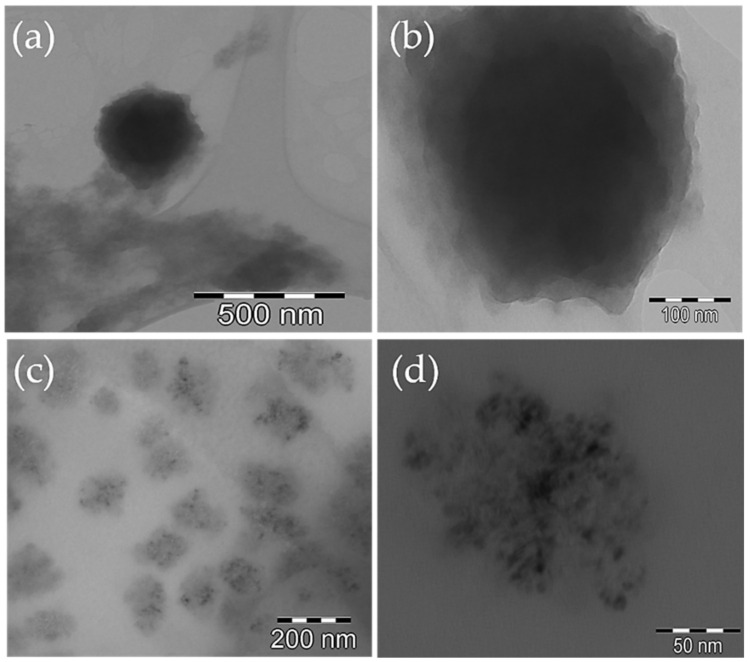
TEM pictures of CS/HY NPs before (**a**,**b**) and after (**c**,**d**) lyophilization at different magnifications.

CS/HY NPs were characterized for morphology by TEM analysis, before and after lyophilization. The obtained pictures, shown in Figure 2, demonstrated the presence of spherical NPs without signs of aggregation. Before lyophilization, the NPs appear fluffy (Figure 2a,b), probably due to a high hydration level, and show a different density between the surface and the matrix, probably because of the presence on the surface of CS and HY chains not totally interacting. After lyophilization (Figure 2c,d), the architecture of the matrix appears inhomogeneous and characterized by areas of higher density and areas of lower density. This could highlight the random alternation of CS and HY chains, which interact in a different extension.

**Figure 3:**
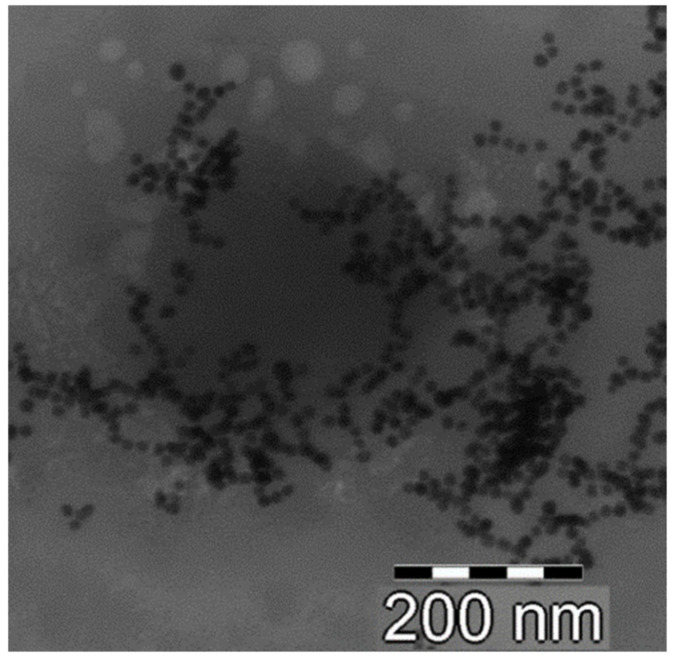
TEM picture of CS/HY NPs after incubation with a colloidal suspension of Au nanoparticles for 1 h.

In order to confirm this hypothesis, we performed TEM analysis of CS/HY NPs after incubation for 1 h with Au colloidal suspension. As shown in Figure 3, an irregular deposition of Au nanoparticles on CS/HY NPs was revealed, demonstrating areas on the surface characterized by a different charge density.

### 3.2. Molecular Characterization

µ-Raman analysis was performed to characterize how HY and CS interact with each other during NP formation. The used approach, already successfully applied for similar systems [37], consisted of monitoring the spectral changes induced by the activation of some interaction in the vibrational bands ascribed to specific functional groups taking part in the interaction itself.

**Figure 4:**
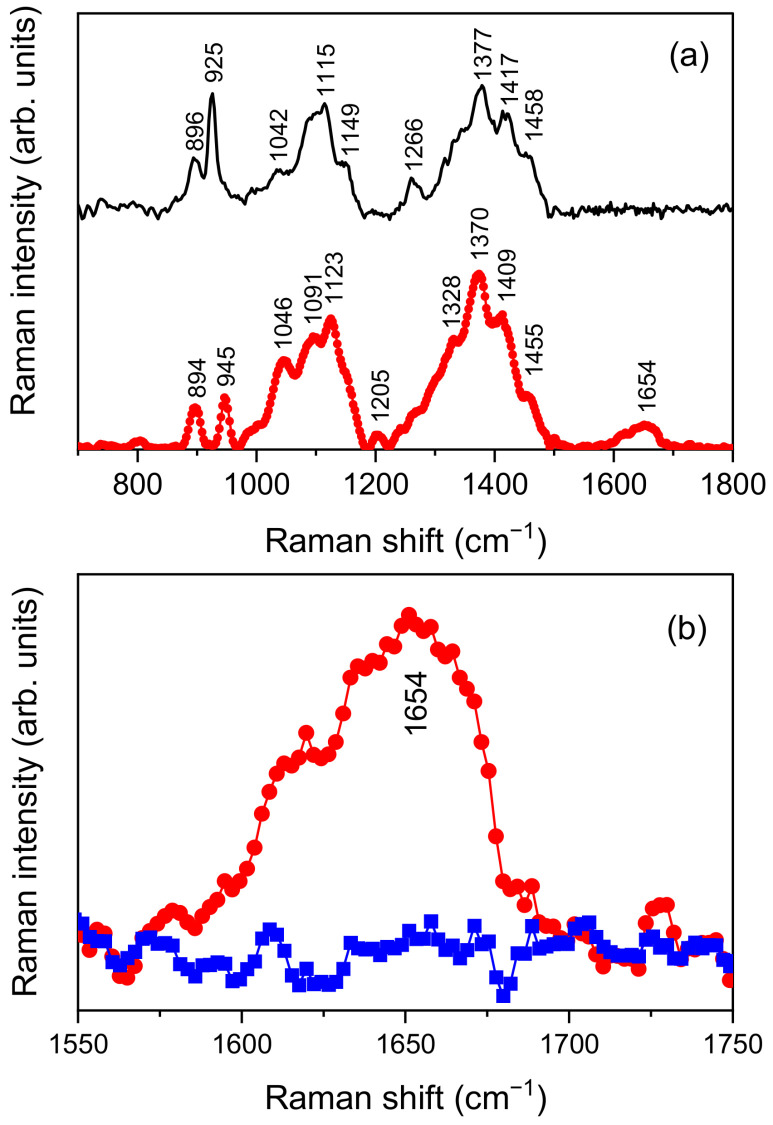
(**a**) µ-Raman experimental spectra, in the 700–1800 cm^−1^ range, of HY (red circles) and CS (black line). (**b**) Comparison, in the 1550–1750 cm^−1^ range, of the Raman experimental spectra of HY (red circles) and CS/HY NP systems (blue squares).

Figure 4a shows the experimental µ-Raman spectra collected for HY and CS in the 700–1800 cm^−1^ spectral range, to which the main vibrational features of both systems belong.

As far as HY is concerned, comparison with literature [38,39] allowed us to identify the peaks at ~894 cm^−1^ and ~945 cm^−1^ as resulting from out-of-plane -CH deformations. The large band from ~970 cm^−1^ to ~1180 cm^−1^ results from the overlapping of at least three different peaks, respectively ascribed to C-C and C-O stretching modes (~1046 cm^−1^), C-OH bending mode of an acetyl group (~1091 cm^−1^), and C-OH and C-H bending modes (~1123 cm^−1^). CH_2_ twisting vibrational modes are responsible for the small band observed at ~1205 cm^−1^. Continuing onward, the wide band in the 1225–1500 cm^−1^ range also appears as the overlapping of four main contributions. The first one, peaked at ~1328 cm^−1^, is an amide III band due to *cis* arrangement of the C=O and N-H functional groups with respect to the C-N bond. Other two, centered at ~1370 cm^−1^ and ~1455 cm^−1^, account for asymmetric and symmetric CH_3_ bending vibrational modes, respectively. The last band, at ~1409 cm^−1^, results from the symmetric COO^−^ stretching mode. Finally, the band at ~1654 cm^−1^ is mainly associated with C=C and amide I C=O vibrations, with low-frequency shoulders deriving from asymmetric COO^−^ stretching vibrational modes.

In the case of CS, based on previous published papers [40], the peaks detected at ~896 cm^−1^ and ~925 cm^−1^ were assigned to -CH out-of-plane deformations; the three main features contributing to the large band in the 980–1180 cm^−1^ range were ascribed to C-C and C-O stretching (~1042 cm^−1^), C-O-C asymmetric stretching (~1115 cm^−1^), and -CN stretching (~1149 cm^−1^) vibrational modes, respectively. Continuing onward, -CH in plane deformations are responsible for the peak detected at ~1266 cm^−1^, whereas -CN stretching justifies the peak at ~1377 cm^−1^, and -CH asymmetric deformations and -CH_2_ in planes bending the peaks at ~1417 cm^−1^ and ~1458 cm^−1^.

As highlighted by an inspection of Figure 4a, the previously described band associated with C=C, C=O and COO^−^ stretching vibrations of HY falls in a spectral range extending from 1550 cm^−1^ to 1750 cm^−1^, independently from contributions ascribable to CS. As a consequence, any change detected in this range by a comparison of the µ-Raman spectra collected for HY and CS/HY NPs can furnish direct evidence of the establishment of interactions involving these functional groups. This comparison is reported in Figure 4b. It evidences the flattening of the experimental band of HY at ~1654 cm^−1^, which testifies to a hindering of the aforementioned vibrational modes resulting from interactions activated during NP formation.

### 3.3. Characterization of CS/HY NPs Loading VBL

**Figure 5:**
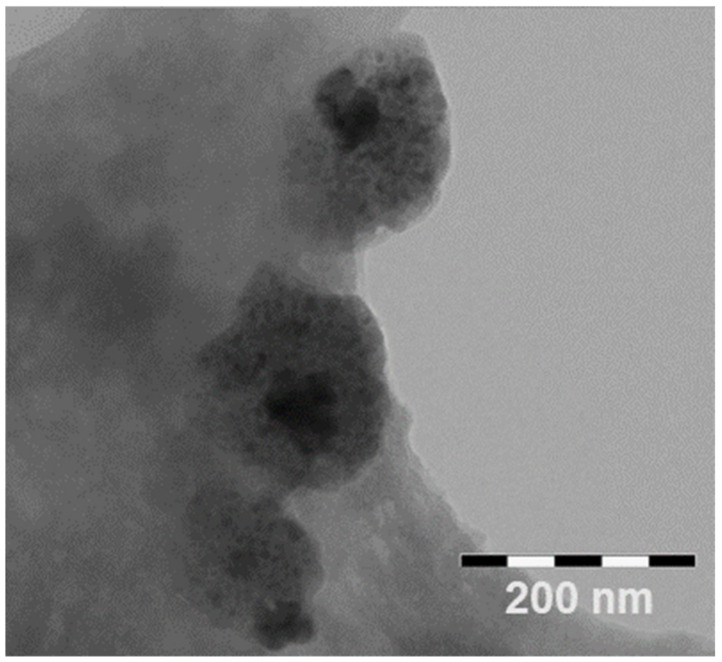
TEM picture of lyophilized 15% VBL-CS/HY NPs.

CS/HY NPs loading VBL (VBL-CS/HY NPs) were prepared adding different amounts of VBL (5, 15 and 20% *w/w* in polymers) to CS solution. As reported in Table 3, in all cases we obtained homogeneous NPs of increasing size by increasing the VBL theoretical amount. Encapsulation efficiency also increases with the increase in VBL until an amount of 15%. Further increases of the drug produced a diminishing of the cargo capability of the system. A less negative zeta potential value was observed with respect to empty NPs. Probably, a partial superficial localization of VBL produced a reduction in negative charge density due to HY. TEM images of VBL-CS/HY NPs depicted the same morphology already observed for empty NPs. The picture of the lyophilized sample is shown in Figure 5.

**Figure 6:**
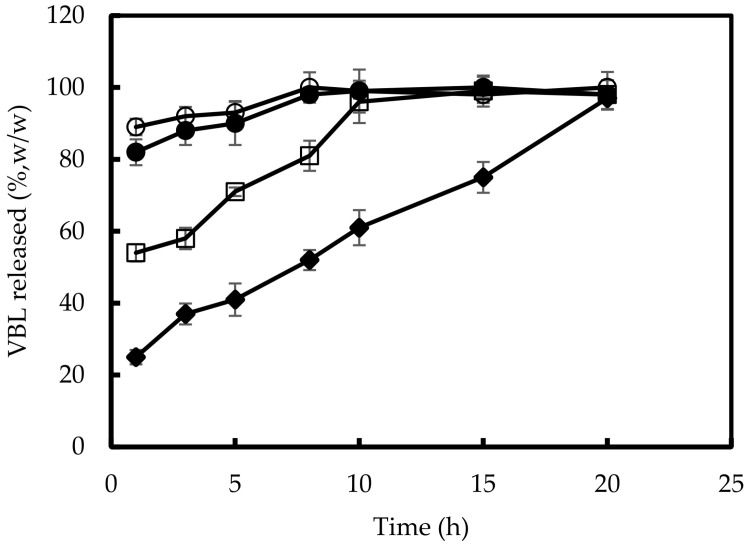
Release profile of VBL from 15% VBL-CS/HY NPs as a function of time and different pH values. Open squares: pH = 5; closed diamonds: pH = 7.4. In addition, the diffusion of free VBL at pH = 7.4 (closed circles) and at pH = 5.0 (open circles) are reported. See text for details. Results are presented as the means of six different experiments ± S.D.

In vitro release studies of VBL from the NPs were performed in PBS at pH 7.4 and pH 5.0 on the formulation prepared with the 15% VBL theoretical amount, due to the best properties in terms of size, E.E. and D.C. showed by this sample. The obtained profile is shown in Figure 6. At neutral pH, we observed a burst effect of about 20% (*w/w*) in the first hour of the experiment that could be a result of the fast release of VBL located on the surface of NPs. After the burst effect, a sustained release of the drug from the NPs was observed with a quantitative drug release within 20 h that, as below demonstrates, is perfectly compatible with the time needed for the internalization of the NPs within K-562 cells. A different profile was observed at acidic pH, consisting of a burst effect of about 60% (*w/w*) in the first hour, and a quantitative release of the drug from the NPs within 10 h from the beginning of the experiment. These results can be expected, considering that the acidic environment can improve the water solubility of the drug with respect to neutral pH, producing more rapid release. A quantitative diffusion of the free drug was observed within 8 h, both at pH = 7.4 and pH = 5.0.

### 3.4. In Vitro Cytotoxic Activity

**Figure 7:**
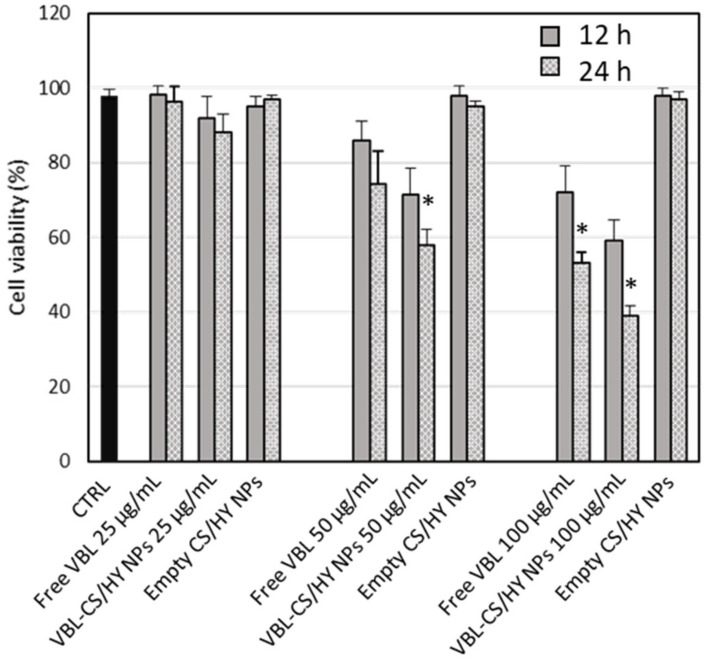
Dose-dependent cytotoxic effect of free VBL and VBL-CS/HY NPs against K-562 cells at different exposition times and doses. Drug cytotoxic effect is expressed as percentage of cellular viability. CTRL accounts for untreated cells, and showed a cell viability always ≥97%. Results are presented as the means of three different experiments (6 replicates for each point) ± S.D. * *p* < 0.05 versus free VBL.

To evaluate the anticancer effect of free VBL and VBL-CS/HY NPs, in vitro studies were performed on K-562 human myeloid leukemia cells as a function of both drug concentration (25, 50 and 100 μg/mL) and time exposition (12 and 24 h). As observed in Figure 7, no cytotoxic effect was exerted by empty CS/HY NPs at all concentrations and times considered. Free VBL showed a dose-dependent and time exposition-dependent cytotoxic effect. About 40% cell mortality was observed after the treatment with 100 μg/mL dose. VBL-CS/HY NPs allowed a significant improvement in the anticancer activity against K-562 cells, showing an effective anticancer activity after just 12 h incubation at a dose of 50 μg/mL, higher than that observed for the free drug at the highest dose. By increasing the exposition time, a more intense cytotoxic effect was observed, and at a dose of 100 μg/mL about 60% cell mortality was registered for the NP formulation. The increase in the cytotoxic effect of VBL loaded-NPs can be explained by a greater and more rapid cellular entrance of the delivered anticancer agent with respect to the free drug, as a consequence of the nanocarrier uptake by K-562 cells, as demonstrated by the internalization studies described in the following.

### 3.5. Internalization Studies

Internalization studies were carried out on K-562 human myeloid leukemia cells. Being the initial hypothesis of the selectivity of CS/HY NPs based on the role played by CD44 in cellular internalization, CD44 expression levels in the used cell line were verified by flow cytometry labeling K-562 cells with FITC-conjugated anti-CD44 antibodies. The images reported in the Supplementary Materials (Figure S1) showed high expression of CD44 on cell surfaces compared to the isotype control, demonstrating the suitability of K-562 for the internalization studies of CS/HY NPs.

The studies were performed on CS/HY NPs loading a bacterial lysate of *L. reuteri* labeled with a fluorescent molecule (labeled NPs). The efficiency of the labeling of the microorganism, before and after lysis, was demonstrated by flow cytometry, and the obtained results are shown in the Supplementary Materials (Figure S2). Furthermore, encapsulation of labeled bacterial lysate by the NPs was confirmed by flow cytometry analysis (Figure S3).

Labeled NPs were characterized as far as size and surface electric properties are concerned. Insignificant variation with respect to empty CS/HY NPs was observed, with a marginal increase in R_H_ (from 110 nm to 125 nm), and an almost unchanged ζ value (from −13.51 ± 1.85 mV to −14.21 ± 0.65 mV for empty NPs and labeled NPs, respectively).

The internalization experiments were performed by co-culturing K-562 cells with labeled NPs, or unlabeled NPs as negative control, and analyzing them with flow cytometry at three different time points (1 h, 6 h and 24 h). Furthermore, in order to saturate the CD44 membrane receptor and to clarify whether the mechanism of NP internalization is exclusively related to the interaction with the receptor or involves some other process, another experiment was performed. In it, before contact with labeled NPs, K-562 cells were incubated for 1 h with free HY in an amount 10 times higher than that used to prepare the NPs.

**Figure 8:**
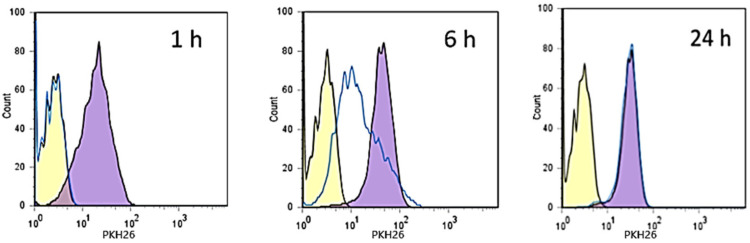
Kinetic of internalization of labeled NPs in K-562 cell lines. Yellow histogram: negative control; violet histogram: K-562 cells treated with labeled NPs; blue line histogram: K-562 cells pre-incubated with free HY for 1 h, washed with PBS, and then treated with labeled NPs.

As observed in Figure 8, a fast internalization was observed for labeled NPs in non-pretreated cells. The uptake is complete within 6 h from the beginning of the experiment. The pretreatment of cells with HY produced a delay in the entry of the NPs into K-562 cells, showing a quantitative internalization within 24 h. CD44 is a saturable receptor [41], and is the main agent responsible of HY turnover [42]. After binding to the membrane receptor, HY, as a CD44-HY complex, is internalized with a mechanism that does not involve *caveolae* or chathrin-coated pits [43,44], but appears to be associated with lipid rafts or protein complexes ankyrin [45] and integrins [46].

**Figure 9:**
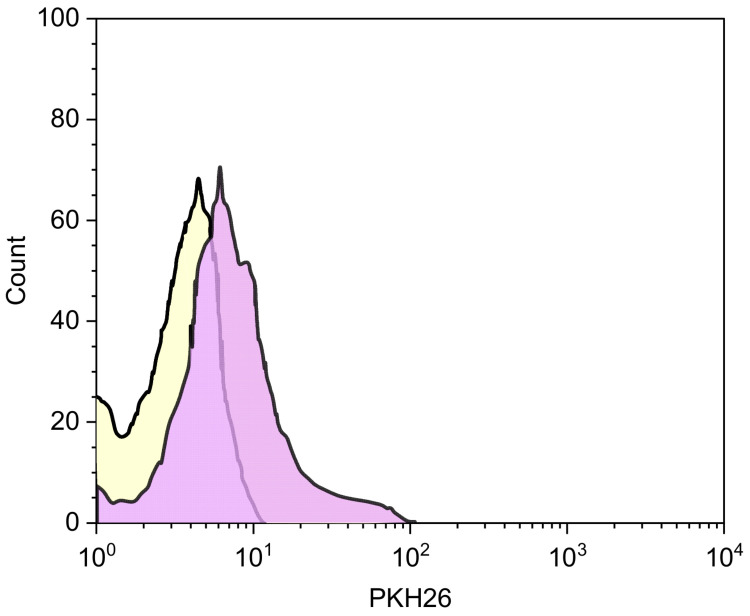
Cytofluorimetric analysis of CD44 negative ZR-75-1 cells incubated with CH/HY NPs for 24 h. Yellow histogram: negative control; violet histogram: ZR-75-1 cells incubated with labeled NPs.

It is conceivable that CD44 receptors present on K-562 membrane cells were saturated by contact with free HY chains, avoiding in this way their interaction with labeled NPs. It is required that the complex HY-CD44 is internalized within the cells [42] and HY degraded by hyaluronidase [47] to permit the release of CD44 and its interaction with labeled NPs. Reasonably, this process produces a delay in the internalization of the labeled NPs. However, the presence of other mechanisms could not be excluded. For this reason, to further confirm the CD44 receptor-mediated internalization of CH/HY NPs, we performed experiments employing CD44-negative human breast cancer cell lines (ZR-75-1) co-cultured for 24 h, with labeled NPs. The results obtained by cytofluorimetric analyses are shown in Figure 9. Very low levels of NP internalization were measured, thus confirming that CD44 expression on cell surfaces plays a decisive role in cellular uptake of HY-based NPs.

The internalization of labeled NPs into K-562 cells was also studied by imaging flow cytometry. By applying a *gate* on the single live cells and selecting on the Ch01 channel the brightfield, on the Ch03 channel the cells treated with labeled NPs, then overlapping the images of the two channels (Ch01/Ch03), it was possible to highlight the localization of the NPs inside the cells, excluding any surface fluorescence resulting from the presence of the NPs on the cell membrane but not linked to their internalization.

**Figure 10:**
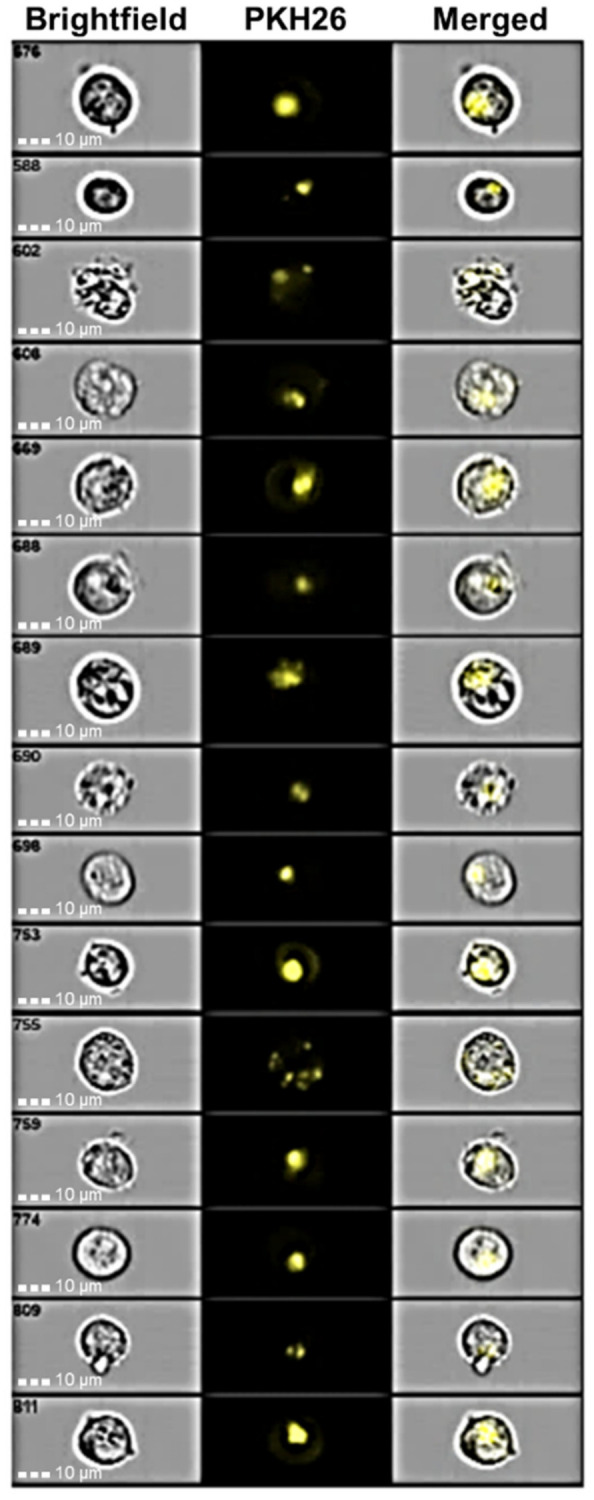
Image cytometry of K-562 treated for 6 h with labeled NPs.

K-562 cells were co-incubated with labeled NPs and analyzed after 6 h. The obtained images, shown in Figure 10, demonstrate high fluorescence intensity for all analyzed cells, highlighting the ability of the NPs to be efficiently internalized.

**Figure 11:**
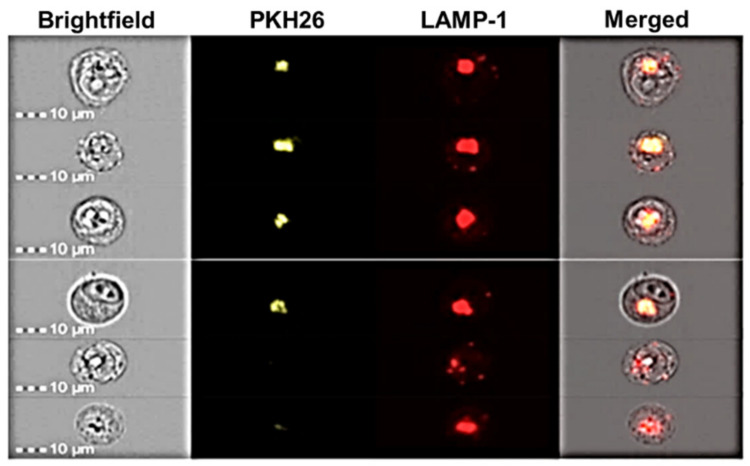
Image cytometry of K-562 cells intracytoplasmic labelled with Alexa fluor 647-conjugated anti-human CD107a (LAMP-1) antibody and treated with labelled CH/HY NPs.

To evaluate the localization of the internalized NPs, experiments were performed using K-562 cells previously intracytoplasmic labeled with Alexa fluor 647-conjugated anti-human CD107a (LAMP-1) antibody. CD107a is a glycoprotein expressed on lysosome membranes, and its linkage with the fluorescent antibody permits identification of lysosomes within the cells. Labeled cells were co-incubated with labeled NPs for 6 h, and the obtained images are shown in Figure 11.

We observed overlap of the fluorescence as a result of labelled NPs with that of the K-562-labelled lysosomes. These results clearly demonstrate the localization of NPs within the lysosomes. Based on previous studies [48], we can hypothesize that CS/HY NPs in lysosomes are degraded, and the encapsulated drug is released within the cytosol. In this way, high amounts of a cytotoxic drug can be presented directly into cancerous cells, increasing its therapeutical efficacy, and reducing side effects. Other studies will be performed to confirm this hypothesis.

## 4. Conclusions

In this study, CH/HY NPs intended for target to cancer cells were prepared according to two different methods, i.e., by ionotropic gelation of CH with TPP, and subsequent coating with HY, or by polyelectrolyte complexation of CH with HY. As revealed by light scattering investigation, NPs produced by coating with HY appear inhomogeneous as a result of the presence in the dispersion of two populations with different sizes. These characteristics are not suitable for the intended use in the therapeutic field.

The CH/HY NPs obtained by polyelectrolyte complexation are spherical, feature a low degree of aggregation, and have optimal sizes (about 100 nm) for cell internalization. They showed negative ζ values, confirming the presence of HY on the surface, as required for the CD44-mediated internalization of CH/HY NPs. A hindering of C=C, C=O and COO^−^ stretching vibrational modes of HY was detected by µ-Raman technique, confirming the establishment of intermolecular interactions between CH and HY upon NP formation.

The CH/HY NPs efficiently encapsulated vinblastine sulfate (VBL), producing no significant variations in size compared to empty NPs, and a slight reduction in ζ value, probably resulting from a partial superficial localization of the positively charged VBL, as demonstrated by the burst effect of about 40% observed in in vitro release studies. A sustained drug release during the following 20 h was observed.

Internalization studies were performed in vitro using a chronic human myeloid leukemia cell line (K-562), and showed significant ability of CH/HY NPs to bind CD44 transmembrane receptors expressed on K-562 cell surfaces. The NPs were totally internalized within 6 h, as demonstrated by ImageStream flow cytometry, while pretreatment of K-562 cells with free HY to saturate CD44 receptors produced a delayed entrance within the cells; this demonstrated the important role played by CD44 in the uptake of CH/HY NPs. The fast uptake of CH/HY NPs observed in the in vitro internalization studies well correlates with the drug release profile from the NPs, and permits us to hypothesize that a therapeutic amount of VBL-loaded CH/HY NPs reaches K-562 cells; this asserts that there is only its anticancer effect reducing the side effects.

Although more studies on the mechanisms of NP internalization are needed, our results suggest that CH/HY NPs could be a potential delivery system for VBL to target cancer cells, reducing the side effects of conventional therapies.

## Figures and Tables

**Table 1:** Composition of CS/TPP NPs.

Samples	CS (mg)	TPP (mg)
1	4	1
2	4	2
3	4	4
4	3	1
5	2	1
6	1	1

**Table 2:** Hydrodynamic radius (R_H_), polydispersity index (P.I.) and zeta potential (ζ) of the analysed CS/TPP NPs (sample 1), CS/TPP-HY NPs (samples 2) and CS/HY NPs (sample 3) after re-suspension of the lyophilized products. S.D.: standard deviation. See text for details.

Samples		mg	R_H_ (nm) ± S.D.	P.I. (%)	ζ (mV) ± S.D.
1	CS	4	180.54 ± 2.89	22 ± 2	+26.54 ± 2.41
	TPP	1			
2	CS	4	162.71 ± 5.41	79 ± 5	−21.85 ± 2.31
	TPP	1			
	HY	0.5			
3	CS	2.82	110.23 ± 3.15	12 ± 3	−13.51 ± 1.85
	HY	0.56			

**Table 3:** Yield, hydrodynamic radius (R_H_), polydispersity index (P.I.), encapsulation efficiency (E.E.), drug content (D.C.) and zeta potential (ζ) of VBL-CH/HY NPs at different VBL amounts after resuspension of the lyophilized products. S.D.: standard deviation. See text for details.

Samples	Yield (%)	R_H_ (nm) ± S.D.	P.I. (%)	E.E. (%)	D.C. (%)	ζ (mV) ± S.D.
Empty NPs	75.02 ± 16.52	110.23 ± 3.15	12 ± 3	--	--	−13.51 ± 1.85
5% VBL-CS/HY NPs	69.74 ± 12.30	115.65 ± 9.87	16 ± 1	37.78 ± 2.89	2.41 ± 5.60	−12.11 ± 0.87
15% VBL-CS/HY NPs	71.00 ± 21.56	122.87 ± 10.22	15 ± 1	56.23 ± 3.54	7.45 ± 3.21	−10.05 ± 0.56
20% VBL-CS/HY NPs	55.78 ± 10.02	330.75 ± 25.89	15 ± 5	18.03 ± 5.27	5.36 ± 9.35	−11.34 ± 0.29

## Data Availability

The data presented in this study are contained within the article and Supplementary Materials.

## Supplementary Materials

The following supporting information can be downloaded at https://www.mdpi.com/article/10.3390/pharmaceutics14050942/s1: Figure S1: Flow cytometry analysis of K-562 cells: (a) isotype control; (b) cells labeled with FITC anti-human CD44 antibody; Figure S2: Cytofluorimetric control of the labeling of *L. Reuteri* with PKH26 and its lysis by sonication. (a) Unlabeled *L. Reuteri*; (b) labeled *L. Reuteri* after four cycles of sonication; Figure S3: Cytofluorimetric analysis of CS/HY NPs loading labeled lysate of *L. reuteri*. Labeled NPs: red histogram; unlabeled NPs: light blue histogram.
