# Supplementary material for: Chitosan-Hyaluronan Nanoparticles for Vinblastine Sulfate Delivery: Characterization and Internalization Studies on K-562 Cells
Source: Pharmaceutics. 2022 Apr 26;14(5):942. doi: 10.3390/pharmaceutics14050942 (PMC9143110; doi:10.3390/pharmaceutics14050942)
Supplement: Supplementary file 1 [file pharmaceutics-14-00942-s001.zip › pharmaceutics-1651034-supplementary.pdf]

SUPPLEMENTARY MATERIAL: Chitosan-Hyaluronan Nanoparticles for Vinblastine Sulfate Delivery: Characterization and Internalization Studies on K-562 Cells

Carmela Cannavà [1,†], Federica De Gaetano [2,†], Rosanna Stancanelli [2], Valentina Venuti [3], Giuseppe Paladini [3,*], Francesco Caridi [3], Corneliu Ghica [4], Vincenza Crupi [3], Domenico Majolino [3], Guido Ferlazzo [1], Silvana Tommasini [2] and Cinzia A. Ventura [2,*]

1 Laboratory of Immunology and Biotherapy, Department of Human Pathology, University of Messina, Via Consolare Valeria, 1, I-98125 Messina, Italy; ccannava@alice.it (C.C.); guido.ferlazzo@unime.it (G.F.)
2 Department of Chemical, Biological, Pharmaceutical and Environmental Sciences, University of Messina, Viale Ferdinando Stagno D'Alcontres 31, I-98166 Messina, Italy; fedegaetano@unime.it (F.D.G.); rstancanelli@unime.it (R.S.); stommasini@unime.it (S.T.)
3 Department of Mathematical and Computer Sciences, Physical Sciences and Earth Sciences, University of Messina, Viale Ferdinando Stagno D'Alcontres 31, I-98166 Messina, Italy; vvenuti@unime.it (V.V.); fcaridi@unime.it (F.C.); vcrupi@unime.it (V.C.); dmajolino@unime.it (D.M.)
4 National Institute of Materials Physics, 405A Atomistilor str., 077125 Magurele-Bucharest, Romania; cghica@infim.ro (C.G.)
* Correspondence: gpaladini@unime.it (G.P.); caventura@unime.it (C.A.V.); Tel.: +39-090-6765463 (G.P.); +39-090-6766508 (C.A.V.)

† First Author.

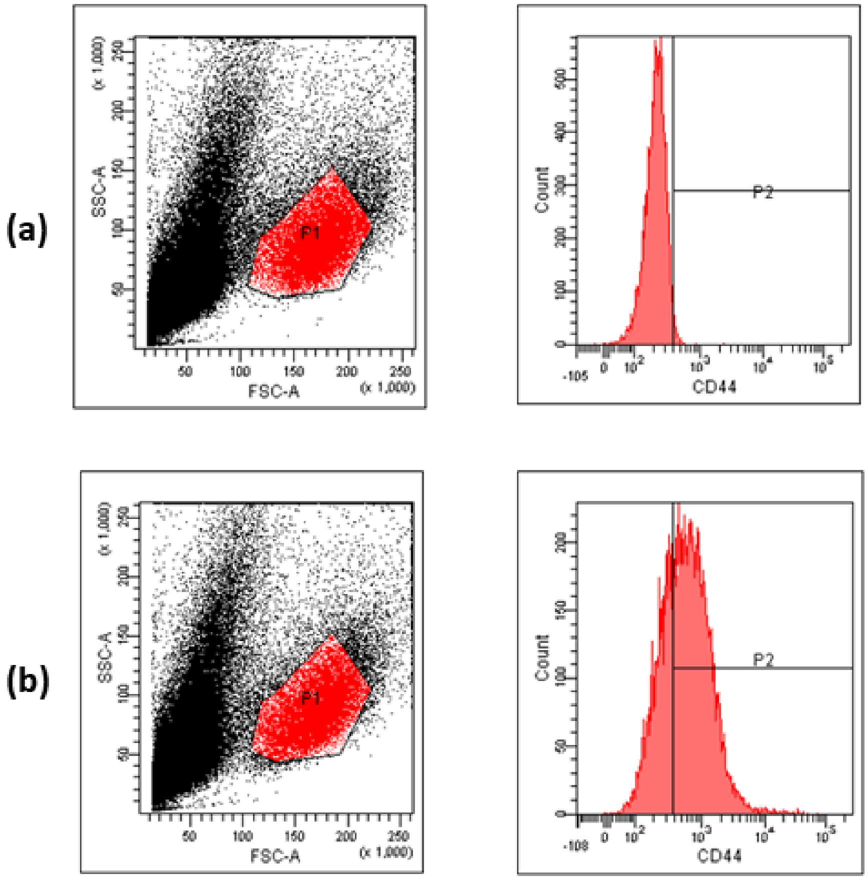

Figure S1. Flow cytometry analysis of K-562 cells: **(a)** isotype control; **(b)** cells labeled with FITC anti-human CD44 antibody.

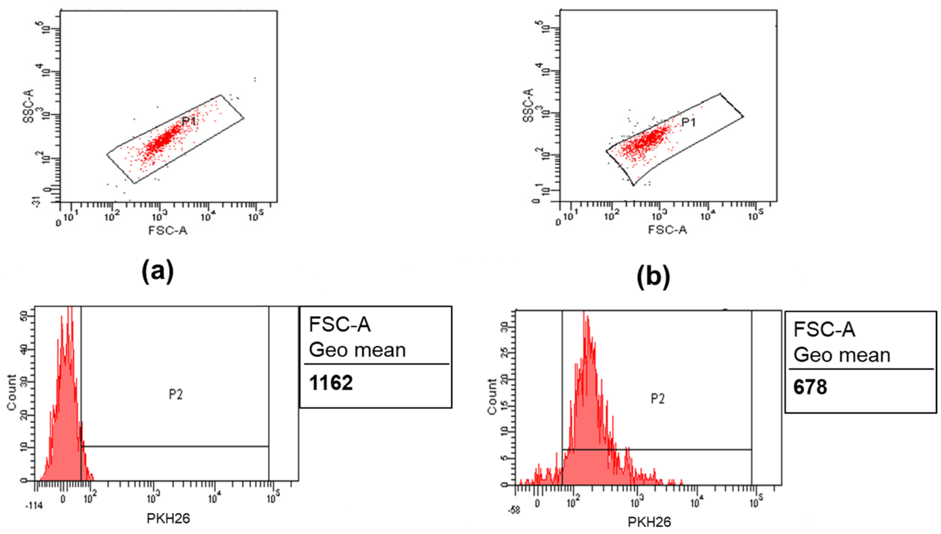

Figure S2. Cytofluorimetric control of the labeling of *L. Reuteri* with PKH26 and its lysis by sonication. **(a)** unlabeled *L. Reuteri*; **(b)** labeled *L. Reuteri* after four cycles of sonication.

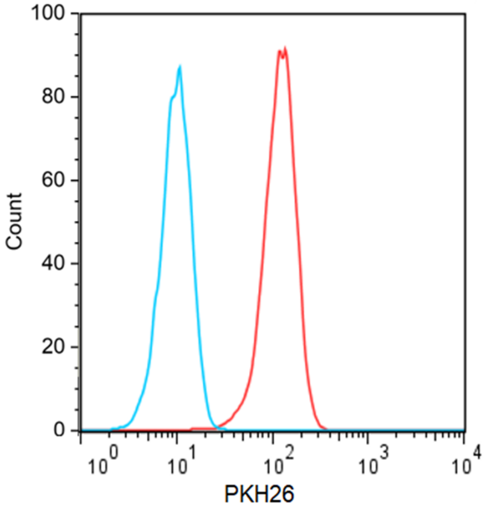

Figure S3. Cytofluorimetric analysis of CS/HY NPs loading labeled lysate of *L. Reuteri*. Labeled NPs: red histogram; unlabeled NPs: light blue histogram.
